# (2*E*)-1-(2,6-Dichloro-3-fluoro­phen­yl)-3-(4-fluoro­phen­yl)prop-2-en-1-one

**DOI:** 10.1107/S1600536812002589

**Published:** 2012-01-25

**Authors:** Richard Betz, Thomas Gerber, Eric Hosten, Aletti S. Praveen, Hemmige S. Yathirajan, Badiadka Narayana

**Affiliations:** a Nelson Mandela Metropolitan University, Summerstrand Campus, Department of Chemistry, University Way, Summerstrand, PO Box 77000, Port Elizabeth 6031, South Africa; b University of Mysore, Department of Studies in Chemistry, Manasagangotri, Mysore 570 006, India; c Mangalore University, Department of Studies in Chemistry, Mangalagangotri 574 199, India

## Abstract

In the title compound, C_15_H_8_Cl_2_F_2_O, the C=C double bond is in the *E* configuration. In the cyrstal, C—H⋯O hydrogen bonds connect the mol­ecules into chains along the *c* axis. A π–π inter­action of 3.628 (1) Å is also observed between two polyhalogenated benzene rings. The dichloro­substituted ring exhibits partial disorder over two sets of sites, with site-occupancy factors of 0.573 (3) and 0.427 (3).

## Related literature

For pharmaceutical background to chalcones, see: Nielsen *et al.* (2004 ▶); Modzelewska *et al.* (2006 ▶); Nowakowska (2007 ▶); Ni *et al.* (2004 ▶). For related structures, see: Yathirajan *et al.* (2006 ▶, 2007 ▶); Betz *et al.* (2011 ▶). For graph-set analysis of hydrogen bonds, see: Etter *et al.* (1990 ▶); Bernstein *et al.* (1995 ▶).
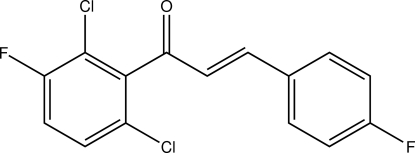


## Experimental

### 

#### Crystal data

C_15_H_8_Cl_2_F_2_O
*M* *_r_* = 313.11
Monoclinic, 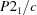

*a* = 12.2311 (3) Å
*b* = 10.3115 (2) Å
*c* = 11.2468 (3) Å
β = 108.935 (1)°
*V* = 1341.70 (6) Å^3^
*Z* = 4
Mo *K*α radiation
μ = 0.50 mm^−1^
*T* = 200 K
0.48 × 0.34 × 0.27 mm

#### Data collection

Bruker APEXII CCD diffractometer
Absorption correction: multi-scan (*SADABS*; Bruker, 2008 ▶) *T*_min_ = 0.825, *T*_max_ = 1.000
12634 measured reflections
3328 independent reflections
2724 reflections with *I* > 2σ(*I*)
*R*_int_ = 0.015

#### Refinement

*R*[*F* ^2^ > 2σ(*F* ^2^)] = 0.035
*wR*(*F* ^2^) = 0.092
*S* = 1.06
3328 reflections
191 parameters
H-atom parameters constrained
Δρ_max_ = 0.24 e Å^−3^
Δρ_min_ = −0.25 e Å^−3^

### 

Data collection: *APEX2* (Bruker, 2010 ▶); cell refinement: *SAINT* (Bruker, 2010 ▶); data reduction: *SAINT*; program(s) used to solve structure: *SHELXS97* (Sheldrick, 2008 ▶); program(s) used to refine structure: *SHELXL97* (Sheldrick, 2008 ▶); molecular graphics: *ORTEP-3* (Farrugia, 1997 ▶) and *Mercury* (Macrae *et al.*, 2008 ▶); software used to prepare material for publication: *SHELXL97* and *PLATON* (Spek, 2009 ▶).

## Supplementary Material

Crystal structure: contains datablock(s) I, global. DOI: 10.1107/S1600536812002589/wn2465sup1.cif

Supplementary material file. DOI: 10.1107/S1600536812002589/wn2465Isup2.cdx

Structure factors: contains datablock(s) I. DOI: 10.1107/S1600536812002589/wn2465Isup3.hkl

Supplementary material file. DOI: 10.1107/S1600536812002589/wn2465Isup4.cml

Additional supplementary materials:  crystallographic information; 3D view; checkCIF report

## Figures and Tables

**Table 1:** Hydrogen-bond geometry (Å, °)

*D*—H⋯*A*	*D*—H	H⋯*A*	*D*⋯*A*	*D*—H⋯*A*
C1—H1⋯O1^i^	0.95	2.51	3.3982 (16)	156
C12—H12⋯O1^i^	0.95	2.55	3.4266 (19)	153

Symmetry code: (i) 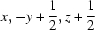.

**Fig. 1.**
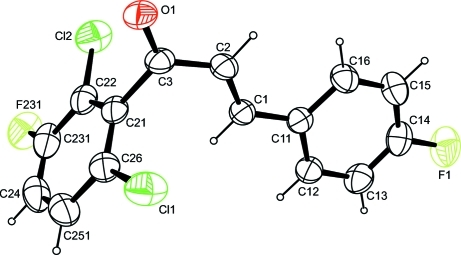
The molecular structure of the title compound, with anisotropic displacement ellipsoids drawn at the 50% probability level. For clarity, only the major component of the disorder model is depicted.

**Fig. 2.**
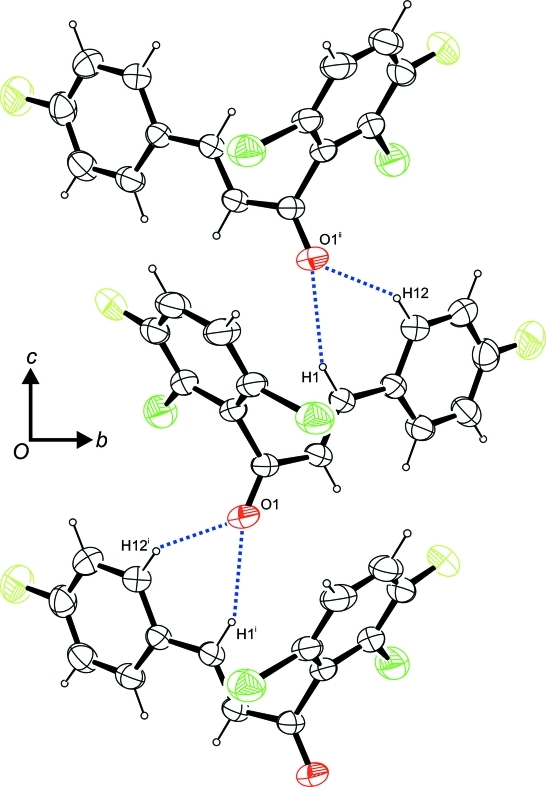
Intermolecular contacts, viewed along [-1 0 0]. For clarity, only the major component of the disorder model is depicted. Symmetry operators: ix, -y + 1/2, z - 1/2; iix, -y + 1/2, z + 1/2. Dashed lines indicate hydrogen bonds.

**Fig. 3.**
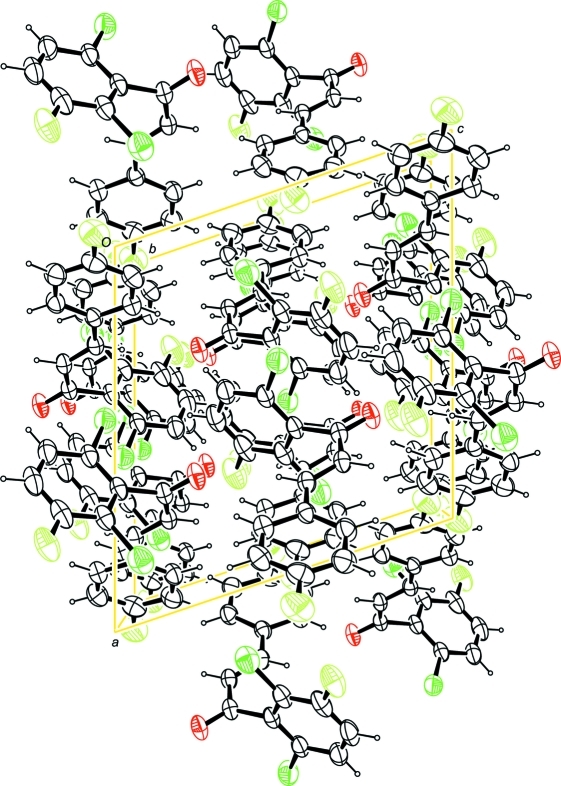
Molecular packing of the title compound, viewed along [0 1 0]. Anisotropic displacement ellipsoids are drawn at the 50% probability level. For clarity, only the major component of the disorder model is depicted.

## supplementary crystallographic information

### Comment

Chalcones constitute an important group of natural products and some of them possess a wide range of biological activities, such as antibacterial (Nielsen *et al.*, 2004) and anticancer (Modzelewska *et al.*, 2006). A review of anti-infective and anti-inflammatory chalcones (Nowakowska, 2007) and recent advances in therapeutic chalcones have been reported (Ni *et al.*, 2004). Related crystal structures of some chalcones, *e.g.* 1-(2,4-dichloro-5- fluorophenyl)-3-(3,4-dimethoxyphenyl)prop-2-en-1-one (Yathirajan *et al.*, 2006) and (2*E*)-1-(2,4-dichlorophenyl)-3-(2-hydroxyphenyl)prop-2-en-1-one (Yathirajan *et al.*, 2007) have been reported. As part of our ongoing studies on chalcones (Betz *et al.*, 2011), the title compound was synthesized and its crystal structure is reported here.

The C═C double bond of the Michael system is in the *E* configuration. The fluorine atom on the polyhalogenated phenyl ring, together with its attached carbon atom is disordered over two sites, as are the ring CH *meta* to it. The site occupancy factors refined to 0.573 (3) and 0.427 (3). The least-squares planes defined by the carbon atoms of the two rings make a dihedral angle of 82.37 (8)° (Fig. 1).

In the crystal structure, intermolecular C—H···O hydrogen bonds are observed (Table 1 and Fig. 2), forming a 6-membered chelate ring. In terms of graph-set analysis (Etter *et al.*, 1990; Bernstein *et al.*, 1995), the descriptor for this pattern is *C*^1^_1_(5)*C*^1^_1_(7) on the unitary level. Molecules are connected into chains along the crystallographic *c* axis. A π–π interaction of 3.628 (1) Å is also observed between two polyhalogenated phenyl rings. The packing of the title compound in the crystal structure is shown in Fig. 3.

### Experimental

To a stirred solution of 1-(2,6-dichloro-3-fluorophenyl)ethanone (1 g, 4.8 mmol) and 4-fluorobenzaldehyde (0.59 g, 4.8 mmol) in ethanol (10 ml), powdered KOH (0.4 g 7.2 mmol) was added at 273 K. The reaction mixture was stirred at room temperature for 1 h. After completion of the reaction, the reaction mixture was poured into ice cold water and acidified with 1.5 N HCl (pH ~3). The resulting precipitate was filtered and dried to afford 1.3 g of the title compound as a pale yellow solid in 86% yield. Single crystals suitable for the diffraction study were grown from a mixture of toluene:acetone (*v*:*v* = 1:1) by slow evaporation at room temperature (m.p.: 421–424 K).

### Refinement

H atoms were placed in calculated positions (C—H = 0.95 Å) and were included in the refinement in the riding model approximation, with *U*_iso_(H) = 1.2*U*_eq_(C).

### Crystal data

**Table:**

C_15_H_8_Cl_2_F_2_O	*F*(000) = 632
*M**_r_* = 313.11	*D*_x_ = 1.550 Mg m^−^^3^
Monoclinic, *P*2_1_/*c*	Melting point = 421–424 K
Hall symbol: -P 2ybc	Mo *K*α radiation, λ = 0.71073 Å
*a* = 12.2311 (3) Å	Cell parameters from 7019 reflections
*b* = 10.3115 (2) Å	θ = 2.7–28.2°
*c* = 11.2468 (3) Å	µ = 0.50 mm^−^^1^
β = 108.935 (1)°	*T* = 200 K
*V* = 1341.70 (6) Å^3^	Block, colourless
*Z* = 4	0.48 × 0.34 × 0.27 mm

### Data collection

**Table:**

Bruker APEXII CCD diffractometer	3328 independent reflections
Radiation source: fine-focus sealed tube	2724 reflections with *I* > 2σ(*I*)
graphite	*R*_int_ = 0.015
φ and ω scans	θ_max_ = 28.3°, θ_min_ = 2.7°
Absorption correction: multi-scan (*SADABS*; Bruker, 2008)	*h* = −16→16
*T*_min_ = 0.825, *T*_max_ = 1.000	*k* = −12→13
12634 measured reflections	*l* = −13→14

### Refinement

**Table:**

Refinement on *F*^2^	Primary atom site location: structure-invariant direct methods
Least-squares matrix: full	Secondary atom site location: difference Fourier map
*R*[*F*^2^ > 2σ(*F*^2^)] = 0.035	Hydrogen site location: inferred from neighbouring sites
*wR*(*F*^2^) = 0.092	H-atom parameters constrained
*S* = 1.06	*w* = 1/[σ^2^(*F*_o_^2^) + (0.0346*P*)^2^ + 0.5003*P*] where *P* = (*F*_o_^2^ + 2*F*_c_^2^)/3
3328 reflections	(Δ/σ)_max_ < 0.001
191 parameters	Δρ_max_ = 0.24 e Å^−^^3^
0 restraints	Δρ_min_ = −0.25 e Å^−^^3^

### Fractional atomic coordinates and isotropic or equivalent isotropic displacement parameters (Å^2^)

**Table:**

	*x*	*y*	*z*	*U*_iso_*/*U*_eq_	Occ. (<1)
Cl1	0.55532 (4)	0.18042 (5)	0.01581 (4)	0.05973 (14)	
Cl2	0.18584 (5)	0.50107 (5)	−0.10590 (5)	0.06391 (15)	
F1	−0.02753 (11)	−0.32385 (11)	−0.03660 (13)	0.0767 (4)	
O1	0.33883 (11)	0.32276 (11)	−0.25385 (9)	0.0498 (3)	
C1	0.23362 (12)	0.10980 (13)	−0.06621 (12)	0.0366 (3)	
H1	0.2685	0.1527	0.0117	0.044*	
C2	0.25296 (12)	0.16144 (14)	−0.16699 (12)	0.0378 (3)	
H2	0.2198	0.1204	−0.2462	0.045*	
C3	0.32232 (13)	0.27733 (14)	−0.16076 (12)	0.0380 (3)	
C11	0.16497 (12)	−0.00496 (13)	−0.06298 (13)	0.0362 (3)	
C12	0.16714 (14)	−0.05235 (15)	0.05394 (14)	0.0435 (3)	
H12	0.2129	−0.0095	0.1282	0.052*	
C13	0.10377 (16)	−0.16073 (16)	0.06360 (16)	0.0522 (4)	
H13	0.1068	−0.1939	0.1434	0.063*	
C14	0.03673 (15)	−0.21884 (16)	−0.04488 (18)	0.0522 (4)	
C15	0.03053 (15)	−0.17524 (16)	−0.16235 (17)	0.0515 (4)	
H15	−0.0173	−0.2177	−0.2358	0.062*	
C16	0.09524 (14)	−0.06845 (15)	−0.17128 (14)	0.0449 (3)	
H16	0.0925	−0.0375	−0.2518	0.054*	
C21	0.37356 (13)	0.34472 (13)	−0.03476 (12)	0.0388 (3)	
C22	0.31594 (15)	0.44789 (15)	−0.00230 (14)	0.0448 (3)	
C24	0.46503 (18)	0.47039 (18)	0.19724 (16)	0.0586 (5)	
H24	0.4956	0.5126	0.2762	0.070*	
C26	0.47854 (14)	0.30709 (15)	0.05110 (13)	0.0434 (3)	
C231	0.36195 (18)	0.50942 (16)	0.11341 (16)	0.0545 (5)	0.573 (3)
F231	0.3031 (2)	0.60294 (18)	0.14524 (19)	0.0723 (7)	0.573 (3)
C251	0.52385 (16)	0.36950 (18)	0.16607 (15)	0.0541 (5)	0.573 (3)
H251	0.5959	0.3423	0.2235	0.065*	0.573 (3)
C232	0.36195 (18)	0.50942 (16)	0.11341 (16)	0.0545 (5)	0.427 (3)
H252	0.3212	0.5795	0.1344	0.065*	0.427 (3)
C252	0.52385 (16)	0.36950 (18)	0.16607 (15)	0.0541 (5)	0.427 (3)
F232	0.6202 (2)	0.3318 (3)	0.2413 (2)	0.0763 (10)	0.427 (3)

### Atomic displacement parameters (Å^2^)

**Table:**

	*U*^11^	*U*^22^	*U*^33^	*U*^12^	*U*^13^	*U*^23^
Cl1	0.0634 (3)	0.0683 (3)	0.0456 (2)	0.0139 (2)	0.01502 (18)	0.00247 (19)
Cl2	0.0770 (3)	0.0586 (3)	0.0613 (3)	0.0189 (2)	0.0295 (2)	0.0032 (2)
F1	0.0894 (8)	0.0584 (7)	0.0933 (9)	−0.0277 (6)	0.0450 (7)	−0.0037 (6)
O1	0.0714 (7)	0.0513 (6)	0.0305 (5)	−0.0051 (5)	0.0216 (5)	0.0038 (4)
C1	0.0447 (7)	0.0352 (7)	0.0304 (6)	0.0025 (6)	0.0129 (5)	−0.0027 (5)
C2	0.0467 (7)	0.0392 (7)	0.0282 (6)	0.0018 (6)	0.0131 (5)	−0.0042 (5)
C3	0.0492 (8)	0.0386 (7)	0.0281 (6)	0.0042 (6)	0.0148 (5)	0.0012 (5)
C11	0.0419 (7)	0.0353 (7)	0.0345 (6)	0.0049 (5)	0.0165 (6)	−0.0011 (5)
C12	0.0533 (8)	0.0427 (8)	0.0367 (7)	0.0006 (7)	0.0178 (6)	−0.0004 (6)
C13	0.0643 (10)	0.0490 (9)	0.0503 (9)	−0.0021 (8)	0.0282 (8)	0.0066 (7)
C14	0.0563 (9)	0.0412 (8)	0.0672 (10)	−0.0065 (7)	0.0313 (8)	−0.0042 (7)
C15	0.0550 (9)	0.0502 (9)	0.0529 (9)	−0.0085 (7)	0.0226 (7)	−0.0146 (7)
C16	0.0520 (8)	0.0474 (8)	0.0379 (7)	−0.0026 (7)	0.0182 (6)	−0.0062 (6)
C21	0.0541 (8)	0.0370 (7)	0.0301 (6)	−0.0070 (6)	0.0202 (6)	0.0002 (5)
C22	0.0640 (9)	0.0382 (7)	0.0395 (7)	−0.0061 (7)	0.0267 (7)	−0.0007 (6)
C24	0.0836 (13)	0.0601 (10)	0.0386 (8)	−0.0307 (10)	0.0289 (9)	−0.0147 (7)
C26	0.0552 (9)	0.0471 (8)	0.0319 (7)	−0.0070 (7)	0.0198 (6)	0.0007 (6)
C231	0.0868 (13)	0.0428 (8)	0.0477 (9)	−0.0151 (8)	0.0407 (9)	−0.0105 (7)
F231	0.1165 (18)	0.0509 (11)	0.0629 (12)	0.0040 (10)	0.0478 (12)	−0.0140 (8)
C251	0.0628 (10)	0.0656 (11)	0.0349 (8)	−0.0214 (8)	0.0175 (7)	−0.0026 (7)
C232	0.0868 (13)	0.0428 (8)	0.0477 (9)	−0.0151 (8)	0.0407 (9)	−0.0105 (7)
C252	0.0628 (10)	0.0656 (11)	0.0349 (8)	−0.0214 (8)	0.0175 (7)	−0.0026 (7)
F232	0.0659 (17)	0.113 (2)	0.0383 (13)	−0.0239 (16)	0.0011 (11)	−0.0047 (13)

### Geometric parameters (Å, °)

**Table:**

Cl1—C26	1.7284 (16)	C13—H13	0.9500
Cl2—C22	1.7284 (18)	C14—C15	1.374 (2)
F1—C14	1.3588 (18)	C15—C16	1.379 (2)
O1—C3	1.2220 (16)	C15—H15	0.9500
C1—C2	1.3410 (19)	C16—H16	0.9500
C1—C11	1.4582 (19)	C21—C26	1.388 (2)
C1—H1	0.9500	C21—C22	1.389 (2)
C2—C3	1.454 (2)	C22—C231	1.392 (2)
C2—H2	0.9500	C24—C231	1.367 (3)
C3—C21	1.5190 (19)	C24—C251	1.373 (3)
C11—C12	1.3950 (19)	C24—H24	0.9500
C11—C16	1.402 (2)	C26—C251	1.389 (2)
C12—C13	1.384 (2)	C231—F231	1.321 (2)
C12—H12	0.9500	C251—H251	0.9500
C13—C14	1.368 (3)		
			
C2—C1—C11	127.18 (13)	C16—C15—H15	120.7
C2—C1—H1	116.4	C15—C16—C11	120.76 (14)
C11—C1—H1	116.4	C15—C16—H16	119.6
C1—C2—C3	123.09 (13)	C11—C16—H16	119.6
C1—C2—H2	118.5	C26—C21—C22	117.71 (13)
C3—C2—H2	118.5	C26—C21—C3	121.98 (13)
O1—C3—C2	121.99 (13)	C22—C21—C3	120.31 (14)
O1—C3—C21	119.41 (13)	C21—C22—C231	120.61 (16)
C2—C3—C21	118.59 (11)	C21—C22—Cl2	120.04 (12)
C12—C11—C16	118.39 (13)	C231—C22—Cl2	119.35 (13)
C12—C11—C1	118.21 (13)	C231—C24—C251	119.29 (15)
C16—C11—C1	123.38 (13)	C231—C24—H24	120.4
C13—C12—C11	121.12 (14)	C251—C24—H24	120.4
C13—C12—H12	119.4	C21—C26—C251	121.18 (15)
C11—C12—H12	119.4	C21—C26—Cl1	120.02 (11)
C14—C13—C12	118.20 (15)	C251—C26—Cl1	118.79 (14)
C14—C13—H13	120.9	F231—C231—C24	119.34 (17)
C12—C13—H13	120.9	F231—C231—C22	119.7 (2)
F1—C14—C13	118.73 (16)	C24—C231—C22	120.88 (16)
F1—C14—C15	118.28 (16)	C24—C251—C26	120.32 (17)
C13—C14—C15	122.98 (15)	C24—C251—H251	119.8
C14—C15—C16	118.52 (15)	C26—C251—H251	119.8
C14—C15—H15	120.7		
			
C11—C1—C2—C3	179.51 (13)	C2—C3—C21—C22	−94.75 (16)
C1—C2—C3—O1	180.00 (14)	C26—C21—C22—C231	−0.9 (2)
C1—C2—C3—C21	−1.2 (2)	C3—C21—C22—C231	179.49 (13)
C2—C1—C11—C12	172.16 (14)	C26—C21—C22—Cl2	179.08 (11)
C2—C1—C11—C16	−9.1 (2)	C3—C21—C22—Cl2	−0.51 (18)
C16—C11—C12—C13	1.1 (2)	C22—C21—C26—C251	0.7 (2)
C1—C11—C12—C13	179.87 (14)	C3—C21—C26—C251	−179.74 (13)
C11—C12—C13—C14	−1.4 (2)	C22—C21—C26—Cl1	−179.36 (11)
C12—C13—C14—F1	−178.78 (15)	C3—C21—C26—Cl1	0.22 (19)
C12—C13—C14—C15	0.7 (3)	C251—C24—C231—F231	177.32 (16)
F1—C14—C15—C16	179.77 (15)	C251—C24—C231—C22	0.3 (2)
C13—C14—C15—C16	0.3 (3)	C21—C22—C231—F231	−176.54 (16)
C14—C15—C16—C11	−0.6 (2)	Cl2—C22—C231—F231	3.5 (2)
C12—C11—C16—C15	−0.1 (2)	C21—C22—C231—C24	0.4 (2)
C1—C11—C16—C15	−178.78 (14)	Cl2—C22—C231—C24	−179.57 (13)
O1—C3—C21—C26	−95.46 (17)	C231—C24—C251—C26	−0.6 (2)
C2—C3—C21—C26	85.68 (17)	C21—C26—C251—C24	0.1 (2)
O1—C3—C21—C22	84.11 (18)	Cl1—C26—C251—C24	−179.89 (12)

### Hydrogen-bond geometry (Å, °)

**Table:**

*D*—H···*A*	*D*—H	H···*A*	*D*···*A*	*D*—H···*A*
C1—H1···O1^i^	0.95	2.51	3.3982 (16)	156
C12—H12···O1^i^	0.95	2.55	3.4266 (19)	153

Symmetry codes: (i) *x*, −*y*+1/2, *z*+1/2.
